# Thirty-Two Years Screening for Diabetic Retinopathy in a Single Centre: An Assessment of Visual Outcomes

**DOI:** 10.3390/medsci14030355

**Published:** 2026-06-28

**Authors:** Francesco Codicè, Tiziana Sanavia, Marina Trento, Elio Striglia, Anatolie Baltatescu, Piero Fariselli, Marcello Montanaro, Massimo Porta

**Affiliations:** 1 AI and Computational Biomedicine Unit, Department of Medical Sciences, University of Torino, 10126 Torino, Italy; francesco.codice@unito.it (F.C.); tiziana.sanavia@unito.it (T.S.); piero.fariselli@unito.it (P.F.); 2 Diabetic Retinopathy Centre, Department of Medical Sciences, University of Torino, 10126 Torino, Italy

**Keywords:** blindness, diabetic retinopathy, prevention, screening, visual function

## Abstract

**Background/Objectives**: Diabetic retinopathy (DR) is a leading cause of preventable visual impairment, and systematic screening is essential to detect sight-threatening stages before symptoms occur. However, long-term real-world evidence on visual outcomes from structured screening programmes remains limited. This study evaluates, in a retrospective analysis of routinely collected clinical data, the real-life visual outcomes of screening for and treating sight-threatening DR, based on 32 years of data collected in a purpose-built centre implementing the 1990 European Working Party recommendations for screening. **Methods**: Screening was performed by retinal photography between 1991 and 2022 in 18,161 patients (63,289 screening episodes). Diabetes specialists graded photographs, referring patients to ophthalmologists when needed. The 10-year trajectories of visual acuity (VA) were assessed in patients with different stages of retinopathy and macular involvement at first screening. **Results**: At first screening, two-thirds of patients had no DR, 15% had mild DR, and the remainder had referable DR or a non-assessable fundus. There was no difference by sex. Patients with more severe DR at first screening had lower VA, but this did not worsen over 10 years. Median VA in 514 patients treated with panretinal photocoagulation for pre-proliferative or proliferative DR changed from 0.10 logMAR (7–8/10) to 0.15 (6–8/10), 1874 ± 1252 days after treatment. In 823 patients photocoagulated for diabetic macular edema, median VA remained 0.10 logMAR (7–8/10) before treatment and 1998 ± 1288 days after treatment. **Conclusions**: Screening for sight-threatening DR using European Working Party recommendations was feasible in everyday practice and was associated with long-term preservation of visual acuity and a low incidence of severe visual loss.

## 1. Introduction

Unless detected and treated before the appearance of symptoms, diabetic retinopathy (DR) may lead to severe visual loss [1]. Consequently, recommendations to screen for asymptomatic sight-threatening DR (STDR) have been issued in most countries [2,3]. However, evidence that screening does prevent loss of vision in the long term is based upon the results of one nation-wide programme associated with reduced incidence of legal blindness in England [4] and few older reports from other countries [5,6,7].

The European Working Party protocol to Screen for DR [8] and its implementation document, the Field Guide-Book [9], were issued in 1990 and validated by independent investigators [10]. This paper reports on cross-sectional and longitudinal data collected over 32 years by implementing the European protocol in a teaching hospital-based screening clinic. Specifically, the study focuses on visual outcomes in patients presenting at their first screening with absent, minimal or more severe DR, and in those undergoing laser photocoagulation as a result of detecting STDR in the process.

## 2. Materials and Methods

### 2.1. Patients

Screening was performed in 18,198 consecutive patients from 1991 through to 2022. Up to the year 2000, data were collected using a purpose-built DOS-based software (SEE—Save Eyes in Europe, v. 1.0) [11], specifically designed to record clinical details according to the European protocol. Afterwards, all data were transferred to a new Windows^®^-based version (SEEw) to overcome problems caused by the “Millennium Bug” [12] which, in the early year 2000, had caused the storage of incongruous data, leading to the records of 37 patients being discarded. The software was designed to close a screening episode only if a full set of pre-defined clinical records (Supplemental Table S1) were successfully collected. In total, 63,289 screening episodes were recorded in 18,161 patients, of which 7920 were females (43.6%).

At first screening, patients were classified according to diabetes type, based on their clinical history and current treatment, as having type 1 diabetes, type 2 diabetes, or insulin-treated type 2 diabetes. Other patients (*n* = 403) included those with Impaired Glucose Tolerance (*n* = 13), those with Impaired Fasting Glucose (*n* = 2), those with diabetes secondary to other conditions (*n* = 299), and those subjected to pancreatic transplant (*n* = 12). Twenty-four patients had gestational diabetes and were monitored at 3-month intervals, while 53 were unclassified.

### 2.2. Procedures and Classification

Up to 22 May in the year 2000, screening was performed by diabetes specialists using direct and indirect ophthalmoscopy and colour photography on 35 mm slide film (Kodak Elite 200 ASA, Eastman Kodak Company, Rochester, NY, USA) using Kowa Pro-I (Kowa Company, Ltd., Nagoya, Japan) and Kowa Pro-II fundus cameras (2237 patients, 5328 episodes). After that date, patients were screened by digital fundus photography (Canon NM45CR, Canon Inc., Tokyo, Japan). Comparison of the two imaging methods showed no significant difference in terms of grading. At least three photographs (anterior chamber with red reflex in mydriasis and two 45° fields, one centred on the macula and the other nasal, including the optic disc at one disc-diameter from the edge of the photograph) were taken by trained medical and nursing personnel, and graded by diabetes specialists. Patients whose retinal photographs were deemed ungradable were referred to an ophthalmologist.

Severity of DR and macular involvement were graded in each eye according to the European protocol [8,9]. Patients with no DR or mild non-proliferative retinopathy that did not require referral (microaneurysms only, isolated hemorrhages and/or cotton wool spots), equivalent to Early Treatment of Diabetic Retinopathy Study (ETDRS) level ≤20 [13], were re-screened in the clinic. Those with moderate non-proliferative DR requiring referral (presence of the above lesions at a higher number and/or in association) or worse such as pre-proliferative, proliferative (PDR), photocoagulated PDR, advanced diabetic eye disease with or without macular involvement, equivalent to ETDRS level >20 [13], previous laser treatment or ungradable pictures, were referred to an ophthalmologist for further assessment and, where necessary, treatment. Macular involvement was defined as the presence of any lesion within 1 disc diameter of the centre of the macula and/or any recent drop in visual acuity that did not improve with pinhole and/or could not be explained by lens or vitreal opacities. To classify patients by DR severity and macular involvement, the worst eye was considered.

Absence/presence of cataract or post-operative intraocular lens implant was defined from patient history and assessment of opacities against the red reflex in photographs of the dilated pupil. Clinical data collected in the course of routine medical visits were anonymized and processed in accordance with the principles of the Declaration of Helsinki as revised in 2008.

### 2.3. Laser Photocoagulation

Laser photocoagulation was applied by one ophthalmologist (MM), as panretinal and/or focal/grid treatment, subject to clinical judgement and according to ETDRS directions for DR and Diabetic Macular Edema (DME) [14,15]. The median time interval between first detection of pre-proliferative or more severe diabetic retinopathy and initiation of laser treatment was 0.1 years (IQR 0.0–1.3).

### 2.4. Prospective Evaluation of Visual Acuity

Visual acuity (VA) was measured in decimals at all visits using optotype projectors or 19″ LCD wall-mounted display monitors with 1280 × 1024 resolution [CSO, Scandicci (Florence, Italy)], ensuring constant light conditions, with the patients’ own lenses and/or pinhole. To assess the evolution of VA over 10 years, patients were first stratified according to the eye with more advanced retinopathy. Subsequently, the eye with the best vision was considered as baseline value.

To treat VA as a continuous variable, decimals were converted to logMAR values [16], such that 0.0 corresponds to 10–9/10; 0.1 to 8–7/10; 0.2 to 6/10; 0.3 to 5/10; 0.4 to 4/10; 0.5 to 3/10; 0.7 to 2/10; 1.0 to 1/10; and 1.3 to 1/20. A value 1.6 was arbitrarily assigned to lower VA (counting fingers, hand movement, light perception, no light perception).

### 2.5. Follow-Up

Yearly follow-up in the same patients was performed as re-screening episodes within multiples of 12 ± 6 months after the first visit. Hence, follow-up screenings were considered at 1 year if they fell within 7–18 months of the first visit, 2 years if within 19–30 months, and so on.

To evaluate the visual outcome of photocoagulation performed following the detection of STDR during screening, VA was assessed retrospectively using the value recorded at the last screening episode before laser treatment and then at all subsequent follow-up visits. The time interval between the start of laser treatment and the last follow-up visit was calculated in years from the corresponding calendar dates. The effects of photocoagulation applied to PDR and DME were analyzed separately, although a number of patients had been subjected to both panretinal and focal/grid treatment over time [14,15].

### 2.6. Statistical Methods

The duration of diabetes in patients with type 1 diabetes was calculated as the interval between the date of diabetes diagnosis and the date of the first screening visit. In patients with type 2 diabetes, the above interval was adjusted by adding 4.39 years, to account for the time during which type 2 diabetes may remain unknown before diagnosis, according to a previously described extrapolation derived from part of the same population [17]. To calculate correlations between VA and severity of DR, retinopathy stages were transformed into numerical values according to ETDRS classification: 10 for absence of DR, 20 for minimal DR, 43 for moderate DR, 53 for pre-proliferative DR, 60 for PDR, 71 for severe/lasered PDR, 80 for advanced diabetic eye disease, and 90 for non-gradable photographs.

Spearman’s rank correlation was applied to assess the associations between ETDRS values of different stages of DR and median VA. Wilcoxon signed-rank test was used to compare VA before and after photocoagulation for both pre-proliferative or proliferative DR and DME detected at screening. In both cases, univariate and then multivariate linear regression models were applied to detect potential risk factors for worsening visual acuity. Backward selection using the Wald test statistic was applied to the multivariate models to consider only the statistically significant factors. Finally, the VA follow-up of these patients was compared to that of a group of controls selected using a matching algorithm. We implemented a one-to-one matching procedure, pairing each case with the most similar control of the same sex by minimizing a standardized distance computed using age at examination and diabetes duration. Individuals with any sign of diabetic retinopathy were excluded from the control pool. This operation was repeated iteratively, removing the selected control patient from the pool of candidates at each step. The same procedure was applied to patients with diabetic maculopathy. Changes in median VA were evaluated up to 10 years following laser treatment. Wilcoxon signed-rank test was applied to compare differences in VA at each year with respect to the baseline, corresponding to the screening visit leading to laser for treated patients and to the first screening episode for controls.

## 3. Results

### 3.1. Prevalence of DR and Macular Involvement at First Screening

Table 1 summarizes the clinical data of all patients at first screening. Overall, 12,281 patients (67.6%) had no DR, 2725 (15.0%) had mild non-referable DR, and 1507 (8.3%) had moderate referable DR or worse. There was no difference in the distribution of retinopathy by sex (χ^2^ = 0.089, *p* = 0.765).

Concerning macular involvement, 1258 patients (6.9%) had lesions within one disc diameter of the centre of the macula, and 373 (2.0%) had already been subjected to grid and/or focal laser treatment. In 198 patients (1.1%) macular lesions suggestive of non-diabetic maculopathy were observed. There was no difference in the distribution of macular involvement by sex (χ^2^ = 3.303, *p* = 0.069).

### 3.2. Visual Acuity in Patients with Different Severity of DR at First Screening and over 10 Years of Follow-Up

**Figure 1:**
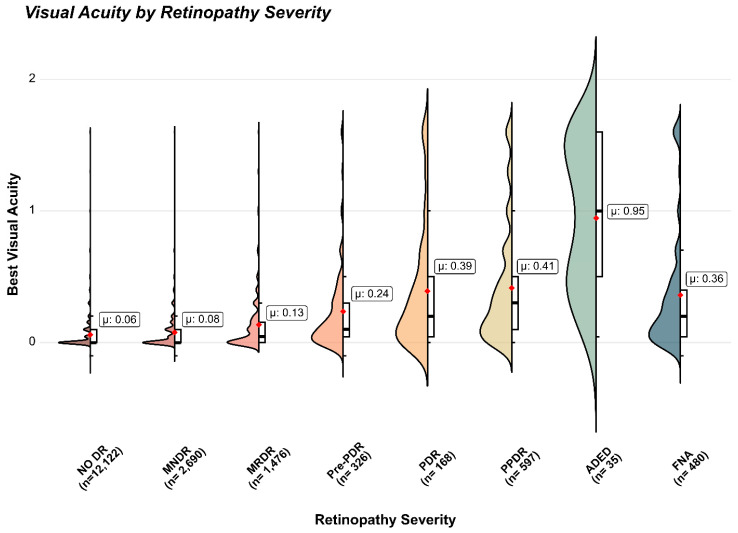
Correlation between severity of DR (*x*-axis), excluding fundus not assessable—far right, and visual acuity (*y*-axis, expressed as logMAR). Spearman r = 0.2393, *p* = 2.151 × 10^−228^; Wilcoxon Rank Sum test comparing non-advanced DR (“NO DR”, “MNDR”) versus more advanced stages (“MRDR”, “Pre-PDR”, “PDR”, “PPDR”, “ADED”): *p* < 2.2 × 10^−16^). Violin and box-plot of VA across severity grades of DR. The bold black line in each box plot indicates the median, whereas the red dot indicates the mean. Legend: DR: Diabetic Retinopathy; MNDR: Mild Non-Referable DR; MRDR: Moderate Referable DR; Pre-PDR: Pre-proliferative DR; PDR: Proliferative DR; PPDR: Photocoagulated PDR; ADED: Advanced Diabetic Eye Disease; FNA: Fundus Not Assessable.

Figure 1 shows the levels of VA in the patients with increasing severity of DR at the first screening. The correlation between the ETDRS stages of DR and the corresponding median VA (excluding non-gradable DR) was r = 0.239 (*p* = 2.151 × 10^−228^). The correlation in type 1 diabetic patients was r = 0.394 (*p* = 2.5 × 10^−109^), while it was r = 0.353 (*p* = 1.94 × 10^−97^) in those with type 2 on insulin, and r = 0.134 (*p* = 3.38 × 10^−44^) in the other patients with type 2. No differences emerged when stratifying by age.

Table 2 shows the levels of VA at baseline and through the following 10 years in patients available for at least one follow-up visit and with different severity of DR at first screening.

Lens opacities were observed in 19.7% of patients with no DR, 29.9% of those with mild DR, 35.8% of moderate DR patients, 37.6% of pre-proliferative DR patients, 45.6% of PDR patients, 52.2% of patients treated by panretinal photocoagulation, and 69.4% of those with Advanced Diabetic Eye Disease. Table 3 and Supplemental Figure S1 show that VA remained below 0.2 logMAR (i.e., above 6/10) up to pre-proliferative DR in patients without lens opacities, but was definitely worse in patients with cataract and those who were subjected to cataract extraction and intra-ocular lens (IOL) implant.

### 3.3. Progression of Visual Acuity in the Patients Treated for Pre-Proliferative and Proliferative DR

Median VA in 514 patients subjected to panretinal photocoagulation in our centre as a result of detecting pre-proliferative or proliferative DR at screening changed from 0.10 logMAR before treatment (corresponding to 7–8/10) to 0.15 (corresponding to 6–8/10) (*p* = 4.017 × 10^−13^), 1874 ± 1252 days after treatment (Supplemental Figure S2).

Considering univariate linear regression, factors associated with VA worsening were age at diagnosis of diabetes (*p* = 0.002) and time after beginning photocoagulation (*p* = 0.02) (Supplemental Table S2). Time course analysis showed VA worsening from 3 years after photocoagulation onwards in patients whose data were available. However, a similar pattern was observed in a control group of 514 matched patients with no DR at first (Supplemental Tables S3 and S4).

On multivariable linear regression, factors independently associated with VA worsening were male sex (estimate and 0.25–97.5% CI: 0.022; 0.001–0.043; *p* = 0.043), age at diagnosis of diabetes (0.047; 0.025–0.069; *p* = 2.98 × 10^−5^) and time since photocoagulation (0.041; 0.019–0.063; *p* = 2.46 × 10^−4^) (Supplemental Table S5).

The time interval between the first detection of pre-proliferative or more severe diabetic retinopathy and initiation of laser treatment (0.1 years; IQR 0.0–1.3) was not significantly associated with post-treatment visual acuity changes at years 1, 2, 5, or 10 (Spearman r ranging from −0.27 to +0.06, all *p* > 0.13).

### 3.4. Visual Acuity in the Patients Treated for Diabetic Macular Edema

Supplemental Table S6 shows median VA values at baseline and over the following 10 years in patients with and without macular involvement at the first screening who were available for follow-up.

Median VA in 823 patients who, over the years, were subjected to photocoagulation following the detection of macular involvement at screening remained 0.1 logMAR (7–8/10) before and after treatment (Supplemental Figure S3). Mean ± SD logMAR values changed from 0.16 ± 0.24 before treatment (corresponding to 8–6/10) to 0.22 ± 0.30 (corresponding to 6–5/10) 1998 ± 1288 days after treatment (*p* = 9.59 × 10^−9^, Wilcoxon test).

In univariate linear regression analysis, the factors associated with VA worsening were age at diagnosis of diabetes (*p* = 0.001) and the time elapsed since beginning laser treatment (*p* = 0.004) (Supplemental Table S7), which remained independent risk factors in multivariable regression analysis (estimate 0.029; 0.25–97.5% CI: 0.014–0.043; *p* = 1.12 × 10^−4^ and 0.026; 0.012–0.041; *p* = 3.83 × 10^−4^, respectively, Supplemental Table S8). Time course analysis showed mild VA worsening from 2 years after photocoagulation onwards in patients whose data were available. However, mild, although significant, deterioration in VA was also observed in a control group of 823 matched patients with no DR at first screening (Supplemental Tables S9 and S10).

The time interval between the first detection of macular involvement and initiation of focal/grid laser treatment (median 0.6 years; IQR 0.1–2.8) was not significantly associated with post-treatment visual acuity changes at years 1, 2, 5, or 10 (linear regression adjusted for baseline visual acuity: all *p* > 0.15).

Among the patients subjected to laser treatment following the detection of STDR at screening, 329 were treated for both PDR and DME.

### 3.5. Progression to Certified Loss of Vision

Seventy-one patients (34% male, mean age at first screening 63.4 ± 11.2 years), developed certified visual loss over a mean follow-up of 8.3 years: partial sightedness (1/20 or 3/60) in 29 and total blindness in 42. Supplemental Table S11 shows the clinical data of these patients at their first and last visits, including the interval between detection of STDR during follow-up and initiation of laser treatment. Of the 20 patients with no DR at first screening, only two developed STDR after 8.3 years, but in neither case was DR the main cause of blindness. In contrast, DR caused visual loss in 8 out of 13 patients who presented with mild retinopathy. Among the 14 patients with moderate referable DR, proliferative retinopathy was the main cause of blindness in 10, and age-related maculopathy in the others. In almost all of the 24 patients who had pre-proliferative DR or worse at first screening, advanced diabetic eye disease was the main cause of blindness despite early detection through detection and treatment.

## 4. Discussion

Despite the urgent need for standardized procedures to screen for STDR and the relevant recommendations issued by scientific societies [2,3], there are few practice-based reports on visual outcomes in patients undergoing large screening programmes. To the best of our knowledge, this is the first study reporting the long-term evolution of visual acuity within a screening programme performed according to a validated [10] set of consensus guidelines [8,9]. Overall, the European Working Party recommendations for DR screening proved applicable to everyday practice and were associated with favourable long-term visual outcomes in patients undergoing screening.

Although issued in 1990, the European Working Party taxonomy for DR is analogous to the simplified classification issued by the American Academy of Ophthalmology in 2003 [18], which is currently in use. In terms of macular damage, however, the European Working Party classification does not discriminate DME into mild, moderate, or severe forms, but implicitly includes within the category “macular involvement” all patients with non-centre and centre involving lesions and those with an otherwise unaccountable drop in vision. These patients were referred for ophthalmological assessment and, where applicable, treatment. However, the term “macular involvement” as used by screeners should not be equated with DME as classified by specialists according to evolving diagnostic criteria and imaging technologies.

Regarding functional outcomes, because few cases of certified blindness are likely to occur in absolute numbers in a screening programme [5,6], monitoring VA trajectories over the years was adopted as a surrogate outcome to assess the effectiveness of implementing the guidelines. Nevertheless, the individual case histories of those patients who became partially sighted or totally blind were also examined.

The ultimate goal of screening is to identify eyes with STDR before symptoms occur, so that treatment (photocoagulation at the time of issuing the European protocols, and much later, intra-vitreal agents) can be applied promptly and appropriately. Although few people with type 1 diabetes progress to blindness if properly screened [5,6], patients with type 2 diabetes may still develop severe visual impairment due to other causes, primarily age-related macular degeneration [19]. In this study, VA decreased with increasing DR severity at first screening (Table 2 and Figure 1). Although intuitive, this is partially in contrast with the notion that DR remains asymptomatic until sight-threatening lesions have fully developed. The increasing prevalence of lens opacities may account, in part, for progressive visual deterioration with worsening DR in our series. In fact, as shown in Table 3, VA was compatible with no or minor visual symptoms up to pre-proliferative DR in the patients with no visible lens opacities. In contrast, vision was worse in patients with cataract and even in those who had undergone cataract extraction, supporting reports that cataract surgery has limited success in eyes with moderate to more severe DR [20,21] and may even result in worsening retinopathy [22]. However, the trajectories of VA over 10 years in patients with different severities of retinopathy at first screening suggest that, while worse DR was associated with lens opacities and lower VA, no further deterioration in vision occurred at any level of retinopathy present upon entry into the programme.

Analysis of the 71 cases that developed partial sightedness or total blindness confirms, on one hand, that age-related macular disease is a major contributor to sight loss in people with no DR at screening, and suggests, on the other, that even mild retinopathy may evolve unfavourably and should be carefully monitored over the years.

In terms of intervention, the programme was able to prevent deterioration in vision in patients in whom STDR was detected at screening. Interestingly, VA in patients who developed STDR and were treated as a result of screening remained better than that measured in patients who developed the same level of retinopathy before their first screening. This might result from being able to follow retinal lesions as they progressed to STDR and intervene before they affected vision, a recognized benefit of a structured screening programme. Although statistically significant, worsening of vision after panretinal photocoagulation was minimal in functional terms, with a drop in visual acuity from 7–8/10 down to 6–8/10, compatible with driving and other daily activities in most countries and substantially better than the expected historical 50% blindness within 5 years in eyes left untreated, according to the Diabetic Retinopathy Study [23]. A report of 10-year visual outcomes in patients referred for proliferative DR detected at screening in the UK found that 7.3% of eyes developed severe visual loss and 14.7% moderate visual loss during follow-up [24]. Also, in the case of DME, a marginal reduction in visual acuity following focal/grid laser was well within the ETDRS-compatible goal of preventing doubling of the visual angle within 5 years [15]. Interestingly, minimal but significant loss of vision was also observed over the same time span in matched controls with no DR, suggesting that at least part of the deterioration in the treated patients may depend on causes other than STDR and/or laser treatment.

Although formally retrospective, all data were prospectively collected using a software that required completion of all information mandated by the European Protocol before an episode could be closed; this study has limitations. First, best-corrected visual acuity was not measured routinely because, by definition, screening is not a full medical visit but a simplified procedure to identify patients at risk. According to the European Working Party recommendations, VA was assessed with patients’ lenses and/or pinhole with the best available equipment to ensure uniform lighting conditions. Secondly, as in 1991 anti-VEGF agents were not yet on the horizon, our software was not designed to collect data on their use. Once available, all eligible patients received intra-ocular anti-VEGF agents but, as injections were not performed in our centre, we could not systematically collect data on the types and modalities of administration.

A further limitation is the progressive attrition of the follow-up cohort over time. As shown in Supplemental Table S12, patients who completed 10 years of follow-up were significantly younger at first screening than those who did not (median 55 vs. 64 years; *p* < 0.0001), suggesting healthy survivor bias. Diabetes duration at first screening was not significantly different between the two groups (median 6 years in both; *p* = 0.17).

In addition, the progressively decreasing sample sizes over time (Table 2) limited advanced approaches to modeling individual trajectories. Therefore, we opted for a simpler, assumption-light approach aimed at providing robust and clinically interpretable population-level trends. Importantly, similar trends were observed in matched control groups, supporting the robustness of the findings.

More importantly, it was not possible to collect data on metabolic control and blood pressure during screening. As pointed out in other studies [16], data on HbA1c and blood pressure are usually not available in a screening setting, unless it caters exclusively to one clinic population. This was not the case in our centre, which provided a screening service to different diabetes units and general practitioners in the area. In addition, before IFCC standardization in 2004 [25], HbA1c results from different laboratories had different reference values. Blood pressure was not measured, but the absence/presence of hypertension was recorded according to the criteria accepted at the time of patient registration and was not found to be a contributing factor to visual outcomes in our population.

Finally, although all patients were given follow-up appointments at the end of each screening session, most did not return regularly (Table 2). Reasons for not attending re-screening appointments may include health reasons, as DR is an established risk factor for cardiovascular and all-cause morbidity and mortality [26], but privacy restrictions prevented us from investigating if disability or death had occurred and for what causes, including socio-economic determinants [27]. This may have introduced a significant selection bias, as the remaining population may have higher compliance, better management of underlying diseases or more severe health conditions, all factors that may facilitate return to follow-up.

## 5. Conclusions

This study suggests that screening for STDR is associated with a low frequency of progression to partial or complete visual loss and with long-term preservation of visual function. Technological advances are expected to make this service available to a larger proportion of the diabetic population, through the increasing use of telemedicine and deep learning-based image analysis [28].

## Figures and Tables

**Table 1:** Clinical data of the patients at first screening and prevalence of non-referable and referable retinopathy, classified by severity. Data shown as percentages and (absolute numbers), unless indicated differently.

	All Patients	Type 1	Type 2	Type 2 + Ins	Other
Number	18,161	3016	11,226	3516	403
Males	56.39 (10,241)	52.4 (1579)	58.4 (6556)	54.1 (1903)	50.4 (203)
Age at diagnosis of diabetes, mean and (SD)		18.8 (13.5)	57.5 (11.8)	51.3 (12.6)	51.1 (16.5)
Age at diagnosis of diabetes, median and (median absolute deviation)		15 (8)	58 (8)	51 (8)	53 (11)
Age at first screening, mean and (SD)		30.9 (17.2)	63.0 (11.0)	64.4 (11.3)	55.5 (14.7)
Age at first screening, median and (median absolute deviation)		29 (13)	64 (7)	65 (8)	58 (9)
Known duration of diabetes at first screening, mean and (SD)		12.5 (10.9)	6.1 (7.6)	13.6 (10.2)	4.9 (7.7)
Known duration of diabetes at first screening, median and (median absolute deviation)		9 (6)	3 (3)	12 (8)	1 (1)
Adjusted duration of type 2 diabetes at first screening, mean and (SD) (Ref. [17])			10.5 (7.6)	18 (10.2)	
Adjusted duration of type 2 diabetes at first screening, median and (median absolute deviation) (Ref. [17])			7.4 (3)	16.4 (8)	
**No DR**	67.6 (12,281)	63.6 (1918)	76.2 (8552)	42.3 (1489)	79.9 (322)
**Mild non-referable DR**	15.0 (2725)	16.3 (493)	13.5 (1514)	19.2 (676)	10.4 (42)
**Referable DR**	17.4 (3142)	20.1 (605)	10.3 (1160)	38.4 (1351)	9.7 (39)
Moderate DR	8.3 (1507)	9.0 (271)	5.3 (593)	17.7 (623)	5.0 (20)
Pre-proliferative DR	1.8 (330)	1.9 (58)	1.1 (118)	4.4 (153)	0.2 (1)
PDR	1.0 (182)	1.7 (50)	0.5 (60)	2.0 (72)	0.0 (0)
Photocoagulated PDR	3.3 (602)	5.6 (169)	1.1 (119)	8.7 (306)	2.0 (8)
Advanced diabetic eye disease	0.2 (36)	0.5 (14)	0.1 (6)	0.4 (15)	0.2 (1)
Macular involvement	6.9 (1258)	7.4 (222)	4.3 (488)	15.4 (542)	1.5 (6)
Photocoagulated DME	2.1 (373)	3.1 (93)	0.8 (85)	5.4 (191)	1.0 (4)
Non-diabetic maculopathy	1.1 (198)	0.2 (5)	1.2 (132)	1.4 (50)	2.7 (11)
**Fundus not assessable**	2.7 (485)	1.4 (43)	2.3 (256)	5.0 (177)	2.2 (9)

DR = Diabetic Retinopathy; PDR = Proliferative Diabetic Retinopathy; DME = Diabetic Macular Edema.

**Table 2:** Median visual acuity (logMAR) over 10 years in patients with different severity of DR at first screening and in those available for at least another follow-up visit.

Year of Follow-Up	n	No DR	Mild Non-Referable DR	Moderate Referable DR	Pre-Proliferative DR	PDR	Lasered PDR	Advanced Diabetic Eye Disease	Fundus Not Assessable
0	18,148	0.08 (n = 12,024)	0.09 (n = 2848)	0.16 (n = 1560)	0.25 (n = 326)	0.44 (n = 177)	0.41 (n = 663)	0.98 (n = 34)	0.36 (n = 516)
1	4255	0.06 (n = 2332)	0.07 (n = 1033)	0.12 (n = 459)	0.27 (n = 68)	0.23 (n = 31)	0.34 (n = 227)	0.92 (n = 6)	0.26 (n = 99)
2	5184	0.05 (n = 3177)	0.07 (n = 1126)	0.11 (n = 478)	0.14 (n = 68)	0.39 (n = 27)	0.29 (n = 209)	0.57 (n = 3)	0.31 (n = 96)
3	4387	0.06 (n = 2698)	0.07 (n = 957)	0.10 (n = 392)	0.21 (n = 49)	0.3 (n = 26)	0.27 (n = 173)	1.0 (n = 3)	0.21 (n = 89)
4	4011	0.06 (n = 2452)	0.07 (n = 874)	0.11 (n = 381)	0.12 (n = 58)	0.34 (n = 21)	0.24 (n = 131)	- (n = 0)	0.32 (n = 94)
5	3461	0.06 (n = 2131)	0.06 (n = 772)	0.08 (n = 315)	0.13 (n = 34)	0.19 (n = 19)	0.24 (n = 121)	0.8 (n = 2)	0.16 (n = 67)
6	3151	0.05 (n = 1893)	0.06 (n = 764)	0.11 (n = 277)	0.23 (n = 33)	0.14 (n = 16)	0.24 (n = 110)	0.2 (n = 3)	0.27 (n = 55)
7	2700	0.06 (n = 1618)	0.07 (n = 655)	0.11 (n = 257)	0.07 (n = 26)	0.29 (n = 13)	0.21 (n = 81)	0.04 (n = 1)	0.19 (n = 49)
8	2382	0.05 (n = 1389)	0.06 (n = 591)	0.09 (n = 227)	0.21 (n = 19)	0.17 (n = 9)	0.22 (n = 99)	0.35 (n = 2)	0.21 (n = 46)
9	2145	0.06 (n = 1217)	0.06 (n = 561)	0.09 (n = 216)	0.1 (n = 22)	0.41 (n = 8)	0.2 (n = 89)	0.2 (n = 4)	0.22 (n = 28)
10	1780	0.05 (n = 1013)	0.06 (n = 461)	0.12 (n = 189)	0.1 (n = 19)	0.1 (n = 10)	0.25 (n = 66)	0.15 (n = 2)	0.23 (n = 20)

**Table 3:** Visual acuity (logMAR) according to absence/presence/operated cataract in patients with different gradings of diabetic retinopathy—ten-year follow-up.

NO CATARACT									
Year of Follow-Up	NO DR	Mild DR	Moderate DR	Pre-Proliferative DR	PDR	Photocoagulated PDR	Advanced Diabetic Eye Disease	Fundus Not Gradable	Total
0	0.04 (n = 8657)	0.04 (n = 1597)	0.06 (n = 746)	0.14 (n = 167)	0.26 (n = 74)	0.24 (n = 150)	0.5 (n = 1)	0.29 (n = 36)	11,428
1	0.03 (n = 1828)	0.03 (n = 596)	0.06 (n = 238)	0.28 (n = 32)	0.31 (n = 21)	0.21 (n = 38)	- (n = 0)	0.31 (n = 10)	2763
2	0.04 (n = 2683)	0.04 (n = 603)	0.08 (n = 237)	0.12 (n = 29)	0.26 (n = 12)	0.2 (n = 36)	0.0 (n = 1)	0.36 (n = 9)	3610
3	0.04 (n = 2227)	0.05 (n = 527)	0.08 (n = 184)	0.17 (n = 26)	0.22 (n = 13)	0.12 (n = 32)	- (n = 0)	0.4 (n = 8)	3017
4	0.04 (n = 2165)	0.05 (n = 478)	0.08 (n = 158)	0.12 (n = 25)	0.28 (n = 8)	0.18 (n = 17)	- (n = 0)	0.62 (n = 5)	2856
5	0.04 (n = 1899)	0.05 (n = 413)	0.07 (n = 141)	0.15 (n = 16)	0.3 (n = 9)	0.07 (n = 21)	- (n = 0)	0.12 (n = 8)	2507
6	0.04 (n = 1715)	0.05 (n = 415)	0.07 (n = 121)	0.17 (n = 16)	0.15 (n = 8)	0.08 (n = 12)	- (n = 0)	0.1 (n = 7)	2294
7	0.04 (n = 1531)	0.05 (n = 334)	0.09 (n = 100)	0.1 (n = 10)	0.35 (n = 10)	0.18 (n = 13)	0.04 (n = 1)	0.15 (n = 4)	2003
8	0.04 (n = 1344)	0.05 (n = 296)	0.11 (n = 90)	0.21 (n = 15)	0.26 (n = 9)	0.18 (n = 14)	- (n = 0)	0.09 (n = 6)	1774
9	0.04 (n = 1221)	0.05 (n = 273)	0.08 (n = 96)	0.14 (n = 18)	0.39 (n = 4)	0.07 (n = 10)	0.0 (n = 1)	0.13 (n = 6)	1629
10	0.04 (n = 1038)	0.06 (n = 205)	0.11 (n = 78)	0.19 (n = 11)	0.4 (n = 4)	0.06 (n = 10)	- (n = 0)	0.32 (n = 5)	1351
**CATARACT**									
0	0.13 (n = 2179)	0.12 (n = 734)	0.21 (n = 484)	0.33 (n = 111)	0.45 (n = 73)	0.46 (n = 255)	1.07 (n = 19)	0.38 (n = 201)	4056
1	0.12 (n = 477)	0.1 (n = 242)	0.21 (n = 120)	0.35 (n = 21)	0.54 (n = 15)	0.49 (n = 49)	0.77 (n = 3)	0.23 (n = 21)	948
2	0.11 (n = 557)	0.12 (n = 221)	0.24 (n = 108)	0.25 (n = 15)	0.72 (n = 13)	0.43 (n = 51)	1.3 (n = 1)	0.14 (n = 26)	992
3	0.11 (n = 520)	0.14 (n = 215)	0.22 (n = 85)	0.55 (n = 15)	0.45 (n = 10)	0.46 (n = 32)	1.0 (n = 1)	0.2 (n = 21)	899
4	0.12 (n = 427)	0.11 (n = 156)	0.25 (n = 68)	0.28 (n = 12)	0.8 (n = 8)	0.36 (n = 35)	- (n = 0)	0.2 (n = 22)	728
5	0.11 (n = 370)	0.14 (n = 128)	0.22 (n = 38)	0.37 (n = 5)	0.43 (n = 5)	0.34 (n = 26)	- (n = 0)	0.13 (n = 18)	590
6	0.11 (n = 335)	0.16 (n = 108)	0.27 (n = 40)	0.38 (n = 8)	0.26 (n = 8)	0.18 (n = 21)	0.3 (n = 1)	0.14 (n = 12)	533
7	0.11 (n = 274)	0.16 (n = 104)	0.26 (n = 39)	0.2 (n = 4)	0.3 (n = 2)	0.29 (n = 13)	- (n = 0)	0.17 (n = 9)	445
8	0.11 (n = 229)	0.13 (n = 79)	0.23 (n = 27)	0.33 (n = 7)	0.3 (n = 4)	0.25 (n = 22)	- (n = 0)	0.17 (n = 14)	382
9	0.11 (n = 196)	0.18 (n = 65)	0.14 (n = 28)	0.5 (n = 1)	0.42 (n = 3)	0.26 (n = 13)	- (n = 0)	0.05 (n = 6)	312
10	0.12 (n = 159)	0.17 (n = 39)	0.22 (n = 29)	0.24 (n = 4)	0.38 (n = 5)	0.35 (n = 11)	- (n = 0)	0.16 (n = 7)	254
**CATARACT EXTRACTION + IOL**									
0	0.1 (n = 1298)	0.13 (n = 365)	0.19 (n = 250)	0.37 (n = 50)	0.6 (n = 25)	0.48 (n = 192)	0.82 (n = 15)	0.36 (n = 243)	2438
1	0.09 (n = 267)	0.09 (n = 103)	0.17 (n = 45)	0.36 (n = 13)	0.2 (n = 4)	0.4 (n = 37)	1.6 (n = 1)	0.29 (n = 41)	511
2	0.08 (n = 341)	0.1 (n = 99)	0.14 (n = 54)	0.42 (n = 6)	0.32 (n = 5)	0.4 (n = 31)	0.5 (n = 2)	0.33 (n = 26)	564
3	0.09 (n = 279)	0.09 (n = 77)	0.16 (n = 44)	0.35 (n = 5)	0.17 (n = 3)	0.39 (n = 23)	0.4 (n = 2)	0.16 (n = 33)	466
4	0.1 (n = 262)	0.12 (n = 65)	0.15 (n = 41)	0.07 (n = 3)	0.1 (n = 3)	0.18 (n = 14)	0.4 (n = 1)	0.26 (n = 23)	412
5	0.09 (n = 228)	0.14 (n = 57)	0.14 (n = 29)	0.05 (n = 2)	0.14 (n = 5)	0.29 (n = 13)	0.2 (n = 1)	0.22 (n = 24)	359
6	0.08 (n = 197)	0.13 (n = 45)	0.09 (n = 24)	0.1 (n = 2)	0.0 (n = 2)	0.48 (n = 14)	0.25 (n = 2)	0.21 (n = 22)	308
7	0.1 (n = 171)	0.07 (n = 34)	0.09 (n = 18)	0.0 (n = 1)	0.25 (n = 2)	0.52 (n = 5)	0.4 (n = 1)	0.22 (n = 14)	246
8	0.08 (n = 150)	0.1 (n = 33)	0.06 (n = 14)	0.0 (n = 1)	0.37 (n = 3)	0.36 (n = 6)	0.5 (n = 1)	0.18 (n = 16)	224
9	0.07 (n = 133)	0.18 (n = 26)	0.14 (n = 16)	0.02 (n = 2)	0.13 (n = 2)	0.46 (n = 7)	0.3 (n = 3)	0.18 (n = 11)	200
10	0.07 (n = 124)	0.13 (n = 21)	0.22 (n = 12)	nan (n = 0)	0.02 (n = 2)	0.55 (n = 7)	0.5 (n = 1)	0.32 (n = 10)	177

## Data Availability

The datasets generated and analyzed in this study are available upon request. The reason for retaining the data presented in this study available on request from the corresponding author is basically that these are clinical data which, although anonymized, we feel should not be made of public domain.

## Supplementary Materials

The following supporting information can be downloaded at: https://www.mdpi.com/article/10.3390/medsci14030355/s1, Figure S1: Visual acuity (logMAR) at first screening by grading of DR and presence/absence of cataract. Figure S2: Boxplot of visual acuity before and 1874 ± 1252 days after photocoagulation for preproliferative or proliferative DR detected at screening. Figure S3: Boxplot of visual acuity before and 1998 ± 1288 days after focal/grid photocoagulation for diabetic macular edema detected at screening. Table S1: Clinical records collected at every screening episode. Table S2: Risk factors for worsening of visual acuity in patients treated by panretinal photocoagulation for preproliferative or proliferative diabetic retinopathy. Table S3: Baseline characteristics of 514 patients treated by retinal photocoagulation for diabetic retinopathy (cases) and 514 matched patients without retinopathy at baseline (controls). Table S4: Mean and Standard Deviation of the visual acuity over 10 years following laser treatment in patients with preproliferative and proliferative DR and in a group of matched controls without retinopathy. Table S5: Risk factors for worsening of visual acuity in patients treated by panretinal photocoagulation for preproliferative or proliferative diabetic retinopathy. Table S6: Median visual acuity (logMAR) from baseline to the following 10 years in patients with and without macular involvement at first screening. Table S7: Risk factors for worsening of visual acuity in patients treated by focal/grid photocoagulation for diabetic macular edema. Table S8: Risk factors for worsening of visual acuity in patients treated by focal/grid photocoagulation for diabetic macular edema. Table S9: Baseline characteristics of 823 patients treated by retinal photocoagulation for diabetic macular edema (cases) and 823 matched patients without retinopathy at baseline (controls). Table S10: Mean and Standard Deviation of visual acuity over 10 years following laser treatment in patients with diabetic macular edema and in a group of matched controls without retinopathy. Table S11: Clinical data of the 71 patients who developed certified blindness, when first screened and when last seen in the Centre. Table S12: Baseline age and diabetes duration in patients who completed ≥10 years of follow-up compared with those who did not.

## Abbreviations

DR	Diabetic Retinopathy
STDR	Sight-Threatening Diabetic Retinopathy
VA	Visual Acuity
ETDRS	Early Treatment Diabetic Retinopathy Study
PDR	Proliferative Diabetic Retinopathy
DME	Diabetic Macular Edema
logMAR	Logarithm of the Minimum Angle of Resolution
MNDR	Mild Non-Referable Diabetic Retinopathy
MRDR	Moderate Referable Diabetic Retinopathy
Pre-PDR	Pre-proliferative Diabetic Retinopathy
PPDR	Photocoagulated Proliferative Diabetic Retinopathy
ADED	Advanced Diabetic Eye Disease
FNA	Fundus Not Assessable
HbA1c	Glycated hemoglobin (hemoglobin A1c), IFCC-standardized
IFCC	International Federation of Clinical Chemistry and Laboratory Medicine
NHS	National Health Service
IOL	Intraocular Lens
SD	Standard Deviation
CI	Confidence Interval
DOS	Disc Operating System
SEE	Save Eyes in Europe (software)
SEEw	Windows^®^ version of SEE
ASA	American Standards Association (film speed rating)
LCD	Liquid Crystal Display
PRIN	Projects of Relevant National Interest (Italian funding scheme)
PNRR	National Recovery and Resilience Plan (Italy)
HPC	High-Performance Computing

## References

[1] Pan Y., Li Y., Cui M., He G., Wang G. (2025). Global, regional and national burden of blindness and vision loss attributable to diabetic retinopathy, 1990–2021: A systematic analysis for the Global Burden of Disease Study 2021. Diabetes Obes. Metab..

[2] Wong T.Y., Sun J., Kawasaki R., Ruamviboonsuk P., Gupta N., Lansingh V.C., Maia M., Mathenge W., Moreker S., Muqit M.M. (2018). Guidelines on diabetic eye care: The International Council of Ophthalmology recommendations for screening, follow-up, referral, and treatment based on resource settings. Ophthalmology.

[3] American Diabetes Association Professional Practice Committee for Diabetes (2026). 12. Retinopathy, neuropathy, and foot care: Standards of Care in Diabetes—2026. Diabetes Care.

[4] Scanlon P.H. (2021). The contribution of the English NHS Diabetic Eye Screening Programme to reductions in diabetes-related blindness, comparisons within Europe, and future challenges. Acta Diabetol..

[5] Agardh E., Agardh C.D., Hansson-Lundblad C. (1993). The five-year incidence of blindness after introducing a screening programme for early detection of treatable diabetic retinopathy. Diabet. Med..

[6] Stefansson E., Bek T., Porta M., Larsen N., Kristinsson J.K., Agardh E. (2000). Screening and prevention of diabetic blindness. Acta Ophthalmol. Scand..

[7] Backlund L.B., Algvere P.V., Rosenqvist U. (1994). New blindness in diabetes reduced by more than one-third in Stockholm County. Diabet. Med..

[8] Retinopathy Working Party (1991). A protocol for screening for diabetic retinopathy in Europe. Diabet. Med..

[9] Kohner E.M., Porta M. (1992). Screening for Diabetic Retinopathy in Europe: A Field Guidebook.

[10] Gibbins R.L., Owens D.R., Allen J.C., Eastman L. (1998). Practical application of the European Field Guide in screening retinopathy by using ophthalmoscopy and 35 mm retinal slides. Diabetologia.

[11] Sivieri R., Rovera A., Porta M. (1995). SEE (Save Eyes in Europe): The London protocol in software. G. Ital. Diabetol..

[12] Remmert K.M. (1999). The millennium bug. Do we have the right antibiotics?. AORN J..

[13] ETDRS Research Group (1991). Grading diabetic retinopathy from stereoscopic colour fundus photographs—An extension of the modified Airlie House classification. ETDRS Report No 10. Ophthalmology.

[14] ETDRS Research Group (1991). Early photocoagulation for diabetic retinopathy. Ophthalmology.

[15] ETDRS Research Group (1995). Focal photocoagulation treatment of diabetic macular edema. Relationship of treatment effect to fluorescein angiographic and other retinal characteristics at baseline: ETDRS report no 19. Arch. Ophthalmol..

[16] Holladay M., Prager T. (1991). Mean visual acuity (letter). Am. J. Ophthalmol..

[17] Porta M., Curletto G., Cipullo D., Rigault de la Longrais R., Trento M., Passera P., Taulaigo A.V., Di Miceli S., Cenci A., Dalmasso P. (2014). Estimating the delay between onset and diagnosis of type 2 diabetes from the time-course of retinopathy prevalence. Diabetes Care.

[18] Wilkinson C.P., Ferris F.L., Klein R.E., Lee P.P., Agardh C.D., Davis M., Dills D., Kampik A., Pararajasegaram R., Verdaguer J.T. (2003). Proposed international clinic diabetic retinopathy and diabetic macular edema disease severity scales. Ophthalmology.

[19] Hansson-Lundblad C., Holm K., Agardh C.D., Agardh E. (2002). A small number of older type 2 diabetic patients end up visually impaired despite regular photographic screening and laser treatment for diabetic retinopathy. Acta Ophthalmol. Scand..

[20] Ridderskär L., Montan P., Kugelberg M., Nilsson I., Lundström M., Behndig A., Zetterberg M. (2022). Outcome of cataract surgery in eyes with diabetic retinopathy: A Swedish national cataract register report. Acta Ophthalmol..

[21] Xia J.L., Patnaik J.L., Lynch A.M., Christopher K.L. (2023). Comparison of cataract surgery outcomes in patients with type 1 vs type 2 diabetes mellitus and patients without diabetes mellitus. J. Cataract Refract Surg..

[22] Tham Y.C., Liu L., Rim T.H., Zhang L., Majithia S., Chee M.L., Tan N.Y.Q., Wong K.-H., Ting D.S.W., Sabanayagam C. (2020). Association of cataract surgery with risk of diabetic retinopathy among Asian participants in the Singapore Epidemiology of Eye Diseases Study. JAMA Netw. Open.

[23] Diabetic Retinopathy Study Research Group (1981). Photocoagulation treatment of proliferative diabetic retinopathy. Clinical application of Diabetic Retinopathy Study (DRS) findings, DRS Report Number 8. Ophthalmology.

[24] Sadiq S.N., Lee C.N., Charmer B., Jones E., Habib M.S., Sandinha M.T., Criddle T., Steel D.H.W. (2024). Referrals for proliferative diabetic retinopathy from two UK diabetic retinopathy screening services: A 10-year analysis of visual outcomes, requirement for vitrectomy, and mortality. Eye.

[25] Hoelzel W., Weykamp C., Jeppsson J.O., Miedema K., Barr J.R., Goodall I., Hoshino T., John W.G., Kobold U., Little R. (2004). IFCC reference system for measurement of hemoglobin A1c in human blood and the national standardization schemes in the United States, Japan, and Sweden: A method-comparison study. Clin. Chem..

[26] Grunwald J.E., Pistilli M., Ying G.S., Maguire M.G., Daniel E., Whittock-Martin R., Parker-Ostroff C., Jacoby D., Go A.S., Townsend R.R. (2021). Progression of retinopathy and incidence of cardiovascular disease: Findings from the Chronic Renal Insufficiency Cohort Study. Br. J. Ophthalmol..

[27] Kelly S.R., Loiselle A.R., Pandey R., Combes A., Murphy C., Kavanagh H., Fitzpatrick P., Mooney T., Kearney P., Crabb D.P. (2021). Factors associated with non-attendance in the Irish national diabetic retinopathy screening programme (INDEAR study report no. 2). Acta Diabetol..

[28] Huemer J., Wagner S.K., Sim D.A. (2020). The evolution of diabetic retinopathy screening programmes: A chronology of retinal photography from 35 mm slides to artificial intelligence. Clin. Ophthalmol..

