# Supplementary material for: Thirty-Two Years Screening for Diabetic Retinopathy in a Single Centre: An Assessment of Visual Outcomes
Source: Med Sci (Basel). 2026 Jun 28;14(3):355. doi: 10.3390/medsci14030355 (PMC13414420; doi:10.3390/medsci14030355)

SUPPLEMENTAL TABLES AND FIGURES

Supplementary Table S1. Clinical Records collected at every screening episode

Progressive Nr	
Date	dd/mm/yyyy
Patient ID	
Gender	□ M □ F
Date of Birth	___/___/_____
Address	
Postal Code	
City of Residence	
**DIABETES**	
Current treatment	□ Diet only<br>□ Diet + oral agents<br>□ Insulin<br>□ Insulin + oral agents
Year of diagnosis of diabetes	_____
Type of diabetes	□ Type 1<br>□ Type 2<br>□ Type 2 on insulin<br>□ Secondary<br>□ IGT<br>□ IFG<br>□ GDM<br>□ Unknown
Cigarette smoking	□ Active<br>□ Stopped for at least 10 years<br>□ Never
Hypertension	□ NO (≤135/8, no treatment)<br>□ YES (≥140/90)<br>□ YES (any value if being treated)
Pregnant	□ NO<br>□ YES
Nephropathy	□ NO<br>□ Microalbuminuria<br>□ Macroalbuminuria<br>□ Renal Failure (eGFR <30 ml/min)<br>□ On Dialisys/Transplant
Certified blindness	□ NO<br>□ YES
Anophtalmus	□ NO<br>□ YES (right eye)<br>□ YES (left eye)
**SCREENING**	
Visual symptoms	□ NO<br>□ YES
Known glaucoma	□ NO<br>□ YES (right eye)<br>□ YES (left eye)
Regular use of eye drops	□ NO<br>□ YES
Lasered following previous screening	□ n.a.<br>□ NO

	□ YES
Visual acuity (…/10)	□ Right eye<br>□ Left eye ______
Using lenses (for distant vision)	□ NO<br>□ YES
Visual acuity improves with pinhole	□ NO<br>□ YES
Pupils dilated	□ NO<br>□ YES
Mydriatic	□ None<br>□ Cyclopentolate<br>□ Tropicamide<br>□ Phenylephrine
Screening test	□ Ophthalmoscopy<br>□ Non-mydriatic fundus photography<br>□ Mydriatic fundus photography
Reactions to mydriatic drops	□ None<br>□ Doubtful<br>□ Redness<br>□ Close Angle Glaucoma<br>□ Other
**SCREENING DIAGNOSIS**	
**Right Eye**	**Left Eye**
Cataract: □ NO<br>□ YES<br>□ Intra-ocular lens<br>□ Not assessable	Cataract: □ NO<br>□ YES<br>□ Intra-ocular lens<br>□ Not assessable
Retinopathy: □ None visible<br>□ Mild (no referral)<br>□ Moderate (referral)<br>□ Preproliferative<br>□ Proliferative<br>□ Lasered proliferative<br>□ Advanced Diab. Eye Disease<br>□ Not assessable	Retinopathy: □ None visible<br>□ Mild (no referral)<br>□ Moderate (referral)<br>□ Preproliferative<br>□ Proliferative<br>□ Lasered proliferative<br>□ Advanced Diab. Eye Disease<br>□ Not assessable
Macular involvement:<br>□ NO<br>□ YES<br>□ Lasered<br>□ Non diabetic Maculopathy<br>□ Not assessable	Macular involvement:<br>□ NO<br>□ YES<br>□ Lasered<br>□ Non diabetic Maculopathy<br>□ Not assessable
Previous laser treatment:<br>□ NO<br>□ For PDR<br>□ For macular edema<br>□ For PDR and edema<br>□ For other reasons<br>□ Not assessable	Previous laser treatment:<br>□ NO<br>□ For PDR<br>□ For macular edema<br>□ For PDR and edema<br>□ For other reasons<br>□ Not assessable
**Quality of photographs**:<br>**Nasal**: □ Good **Temporal**:□ Good<br>□ Sufficient □ Sufficient<br>□ Insufficient □ Insufficient<br>**Centering**:<br>**Nasal** :□ Centred **Temporale**:□Centred<br>□ Partially centred □ Partially<br>□ Not centred □ Not centred	**Quality of photographs**:<br>**Nasal**: □ Good **Temporal**:□ Good<br>□ Sufficient □ Sufficient<br>□ Insufficient □ Insufficient<br>**Centering**:<br>**Nasal** :□ Centred **Temporale**:□Centred<br>□ Partially centred □ Partially<br>□ Not centred □ Not centred

**LESIONS**	
**Right Eye**	**Left Eye**
**Diabetic Retinopathy**:<br>□ Microaneurysms/Microhaemorrhagees<br>□ Cotton Wool Spots<br>□ Spot Haemorrhages<br>□ Flame-Shaped Haemorrhages<br>□ Hard Exudates<br>□ Circinate Hard Exudates<br>□ IRMA<br>□ Venous Abnormalities<br>□ Disc New Vessels<br>□ Peripheral New Vessels<br>□ Previous Laser Therapy<br>□ Vitreous Haemorrhages<br>□ Retinal Detachment	**Diabetic Retinopathy**:<br>□ Microaneurysms/Microhaemorrhagees<br>□ Cotton Wool Spots<br>□ Spot Haemorrhages<br>□ Flame-Shaped Haemorrhages<br>□ Hard Exudates<br>□ Circinate Hard Exudates<br>□ IRMA<br>□ Venous Abnormalities<br>□ Disc New Vessels<br>□ Peripheral New Vessels<br>□ Previous Laser Therapy<br>□ Vitreous Haemorrhages<br>□ Retinal Detachment
**Other Eye Conditions**:	**Other Eye Conditions**:
**New appointment:**	□ Repeat Screening<br>□ Ophthalmic Consultation<br>□ Photocoagulation
**When**:	□ 2 years<br>□ 1 year<br>□ 6 months<br>□ 3 months<br>□ 2 months<br>□ 1 month<br>□ As soon as possible<br>□ Immediately

Supplemental Table S2. Risk factors for worsening of visual acuity in patients treated by panretinal photocoagulation for preproliferative or proliferative diabetic retinopathy. Results from univariate linear regression: coefficients with confidence intervals (CI) and Wald test p-values. Significance: *<0.05, **<0.01

Risk Factors	Coefficient (CI)	P-value
**Male Sex**	0.018 (-0.004 – 0.039)	0.101
**Retinopathy Grading at first screening**	-0.002 (-0.024 – 0.019)	0.832
**Smoking**	-0.017 (-0.039 – 0.004)	0.111
**Adjusted duration of diabetes at first screening**	0.003 (-0.019 – 0.024)	0.810
**Age at diagnosis of diabetes**	**0.034 (0.013 – 0.055)**	**0.002****
**Time after laser treatment**	**0.026 (0.005 – 0.048)**	**0.015***
**Hypertension**	-0.001 (-0.023 – 0.02)	0.906
**Being on insulin treatment**	-0.005 (-0.026 – 0.016)	0.650

Supplemental Table S3: Baseline characteristics of 514 patients treated by retinal photocoagulation for diabetic retinopathy (cases) and 514 matched patients without retinopathy at baseline (controls).

	**Cases**		**Controls**	
	**Mean**	**SD**	**Mean**	**SD**
**Male Sex**	57.00%	—	57.00%	—
**Age**	25.01	28.29	35.84	26.69
**Diabetes duration**	14.97	9.89	11.81	8.99
**Age Diagnosis**	39.40	16.53	39.48	16.48

Supplemental Table S4. Mean and Standard Deviation of the visual acuity over 10 years following laser treatment in patients with preproliferative and proliferative DR and in a group of matched controls without retinopathy. Wilcoxon signed-rank test was applied to compare differences of visual acuity at each year with respect to the baseline. Significance: *<0.05, **<0.01, ***<0.001

**Year of follow-up**	**Lasered**				**Controls**			
	**n**	**mean**	**SD**	**p value**	**n**	**mean**	**SD**	**p value**
Baseline	514	0.18	0.26		514	0.03	0.07	-
1	164	0.27	0.34	0.074	264	0.03	0.06	0.423
2	153	0.27	0.36	0.051	311	0.03	0.07	0.178
3	122	0.26	0.32	0.008**	294	0.04	0.07	4.00E-0.4***
4	105	0.22	0.25	3.00E-0.4***	324	0.03	0.07	0.006**
5	94	0.18	0.23	0.032*	276	0.04	0.07	2.00E-0.4***
6	90	0.22	0.33	3.00E-0.4***	278	0.04	0.08	4.85E-07***
7	84	0.21	0.32	0.019*	278	0.04	0.09	2.14E-05***
8	63	0.22	0.31	0.019*	243	0.03	0.06	0.008**
9	47	0.15	0.17	0.019*	237	0.04	0.08	0.007**
10	38	0.22	0.33	0.001**	227	0.04	0.07	0.001**

Supplemental Table S5. Risk factors for worsening of visual acuity in patients treated by panretinal photocoagulation for preproliferative or proliferative diabetic retinopathy. Results from multivariable linear regression: coefficients with confidence intervals (CI) and Wald test p-values. Significance: *<0.05, **<0.01, ***<0.001

	**Estimate**	**2.5% CI**	**97.5% CI**	**P-value**	**Significance**
**Male sex**	0.022	0.001	0.043	0.043	*
**Age at first screening**	0.047	0.025	0.069	2.98E-05	***
**Time from beginning laser treatent**	0.041	0.019	0.063	2.46E-04	***

Supplemental Table S6 - Median visual acuity (logMAR) from baseline to the following 10 years in patients with and without macular involvement at first screening.

Year of follow up	n	No macular lesions	Macular involvement	Photocoagulated DME	Non diabetic maculopathy	Fundus not assessable
0	18144	0.09 (n=15706)	0.27 (n=1233)	0.36 (n=481)	0.33 (n=206)	0.34 (n=518)
1	4253	0.07 (n=3599)	0.24 (n=280)	0.24 (n=238)	0.21 (n=44)	0.23 (n=92)
2	5182	0.06 (n=4533)	0.19 (n=276)	0.23 (n=232)	0.21 (n=44)	0.28 (n=97)
3	4386	0.06 (n=3858)	0.17 (n=211)	0.23 (n=201)	0.3 (n=37)	0.19 (n=79)
4	4010	0.06 (n=3491)	0.16 (n=224)	0.17 (n=173)	0.16 (n=36)	0.29 (n=86)
5	3460	0.06 (n=3044)	0.14 (n=180)	0.17 (n=144)	0.27 (n=32)	0.14 (n=60)
6	3151	0.06 (n=2796)	0.14 (n=146)	0.18 (n=136)	0.26 (n=21)	0.24 (n=52)
7	2700	0.06 (n=2391)	0.15 (n=128)	0.12 (n=117)	0.16 (n=18)	0.2 (n=46)
8	2382	0.06 (n=2102)	0.13 (n=102)	0.17 (n=123)	0.25 (n=22)	0.2 (n=33)
9	2145	0.06 (n=1882)	0.12 (n=93)	0.13 (n=126)	0.22 (n=17)	0.23 (n=27)
10	1780	0.06 (n=1552)	0.15 (n=100)	0.20 (n=97)	0.18 (n=15)	0.25 (n=16)

Supplemental Table S7. Risk factors for worsening of visual acuity in patients treated by focal/grid photocoagulation for diabetic macular edema. Results from univariate linear regression: coefficients with confidence intervals (CI) and Wald test p-values.

Risk Factors	Coefficient (CI)	P-value
Male Sex	-0.004 (-0.018 - 0.011)	0.620
Retinopathy Grading at first screening	0.0016 (-0.013 - 0.016)	0.826
Smoking	-0.004 (-0.019 - 0.010)	0.558
Adjusted duration of diabetes at first screening	0.002 (-0.013 - 0.016)	0.885
**Age at diagnosis of diabetes**	**0.024 (0.010 - 0.039)**	**0.001**
Hypertension	0.0026 (-0.012 - 0.017)	0.723
**Time after laser treatment**	**0.021 (0.007 - 0.036)**	**0.004**
Being on insulin treatment	-0.006 (-0.020 - 0.009)	0.429

Supplemental Table S8. Risk factors for worsening of visual acuity in patients treated by focal/grid photocoagulation for diabetic macular edema. Results from multivariable linear regression: coefficients with confidence intervals (CI) and Wald test p-values. Significance: ***<0.001

	Estimate	2.5% CI	97.5% CI	P-value	Significance
**Age at first screening**	0.029	0.014	0.043	1.12E-04	***
**Time from beginning laser treatent**	0.026	0.012	0.041	3.83E-04	***

Supplemental Table S9: Baseline characteristics of 823 patients treated by retinal photocoagulation for diabetic macular edema (cases) and 823 matched patients without retinopathy at baseline (controls). N = 823.

	Cases		Controls	
	**Mean**	**SD**	**Mean**	**SD**
**Male Sex**	57.35	—	57.35	—
**Age**	22.26	27.71	34.20	27.41
**Diabetes duration**	13.69	9.34	10.68	8.36
**Age Diagnosis**	40.36	15.87	40.43	15.87

Supplemental Table S10. Mean and Standard Deviation of visual acuity over 10 years following laser treatment in patients with diabetic macular edema and in a group of matched controls without retinopathy. Wilcoxon signed-rank test was applied to compare differences of visual acuity at each year with respect to the baseline. Significance: *<0.05, **<0.01, ***<0.001

**Year of follow-up**	**Lasered**				**Controls**			
	**n**	**mean**	**SD**	**p value**	**n**	**mean**	**SD**	**p value**
Baseline	823	0.16	0.24	-	823	0.05	0.10	-
1	266	0.23	0.33	0.315	505	0.05	0.12	0.374
2	233	0.24	0.36	6.450E-05***	562	0.05	0.08	0.243
3	192	0.20	0.24	0.031*	532	0.07	0.14	4.898E-05***
4	193	0.17	0.25	1.69E-04***	507	0.06	0.14	0.002**
5	153	0.18	0.25	0.005**	459	0.06	0.14	1.08E-04***
6	164	0.15	0.23	3.08E-04***	452	0.07	0.14	4.214E-07***
7	128	0.16	0.26	0.015*	413	0.06	0.13	4.090E-07***
8	130	0.21	0.31	1.90E+06***	368	0.05	0.12	0.009**
9	106	0.12	0.19	0.023*	356	0.06	0.14	6.12E-04***
10	83	0.16	0.27	1.27E-04***	309	0.04	0.08	5.99E-04***

Supplemental Table S11. Clinical data of the 71 patients who developed certified blindness, when first screened and when last seen in the Centre. Abbreviations: DR: Diabetic Retinopathy; M: Males; N: Number of patients; VA: Visual Acuity; IQDR: Interquartile Deviation Range; PDR: Proliferative; Prepr: Pre-proliferative; Mod: Moderate; Not assess: not assessable.

Initial DR grading	N	Gender (M)	Age at 1st screening	VA at 1st screening	Cataract at 1st screening	Final DR grading	Age at last screening	Cataract at last screening	Main cause of visual loss	Time interval from first Moderate DR diagnosis to laser treatment (median - IQDR)
**No DR**	20	4 (20.0%)	64.2±11.1	0.26±0.27	None=9 (45.0%)<br>Yes=7 (35.0%)<br>Operated=3 (15.0%)<br>Not assess=1 (5.0%)	None=14 (70.0%)<br>Mild=3 (15.0%)<br>Mod=1 (5.0%)<br>Prepr=1 (5.0%)<br>PDR=1 (5.0%)<br>Lasered=0<br>Advanced=0	74.2±11.1	None=3 (15.0%)<br>Yes=6 (30.0%)<br>Operated=10 (50.0%)<br>Not assess=1 (5.0%)	Age-related maculopathy=12<br>Cataract=2<br>Severe myopia=2<br>Retinitis pigmentosa=1<br>Not assess.=3	Not Available
**Mild DR**	13	5 (38.5%)	60.2±16.8	0.27±0.36	None=5 (38.5%)<br>Yes=5 (38.5%)<br>Operated=1 (7.7%)<br>Not assess=2 (15.4%)	None=3 (23.1%)<br>Mild=2 (15.4%)<br>Mod=2 (15.4%)<br>Prepr=0<br>PDR=3 (23.1%)<br>Lasered=3 (23.1%)<br>Advanced=0	69.0±17.9	None=3 (23.1%)<br>Yes=7 (53.8%)<br>Operated=3 (23.1%)<br>Not assess=0	Advanced DR=8<br>Age-related maculopathy=2<br>Optic neuritits=2<br>Central causes=1	0.9 (0.0-1.9)
**Moderate (referable) DR**	14	4 (28.6%)	62.7±11.6	0.35±0.33	None=6 (42.9%)<br>Yes=6 (42.9%)<br>Operated=2 (14.3%)<br>Not assess=0	None=0<br>Mild=0<br>Mod=5 (35.7%)<br>Prepr=0<br>PDR=2 (14.3%)<br>Lasered=6 (42.9%)	69.4±11.2	None=4 (28.6%)<br>Yes=5 (35.7%)<br>Operated=5 (35.7%)<br>Not assess=0	Advanced DR=10<br>Age-related maculopathy=4	0.2 (0.0-1.1)

						Advanced=1 (7.1%)					
**Pre-proliferative**	5	1 (20.0%)	66.0±10.1	0.52±0.35	None=2 (40.0%)<br>Yes=2 (40.0%)<br>Operated=0<br>Not assess=1 (20.0%)	None=0<br>Mild=0<br>Mod=0<br>Prepr=1 (20.0%)<br>PDR=0<br>Lasered=4 (80.0%)<br>Advanced=0	69.6±11.2	None=1 (20.0%)<br>Yes=3 (60.0%)<br>Operated=1 (20.0%)<br>Not assess=0	Advanced DR=5	0.4 (0.0-0.8)
**PDR**	6	4	62.2±4.4	0.62±0.24	None=1<br>Yes=5<br>Operated=0<br>Not assess=0	None=0<br>Mild=0<br>Mod=0<br>Prepr=0<br>PDR=1<br>Lasered=5<br>Advanced=0	71.2±6.3	None=0<br>Yes=3<br>Operated=3<br>Not assess=0	Advanced DR=6	0.0 (0.0-0.9)
**Lasered DR**	11	4 (36.4%)	67.1±6.1	0.75±0.30	None=2 (18.2%)<br>Yes=7 (63.6%)<br>Operated=2 (18.2%)<br>Not assess=0	None=0<br>Mild=0<br>Mod=0<br>Prepr=0<br>PDR=1 (9.1%)<br>Lasered=10 (90.9%)<br>Advanced=0	75.6±8.3	None=0<br>Yes=5 (45.4%)<br>Operated=4 (36.4%)<br>Not assess=2 (18.2%)	Advanced DR=10<br>Age-related maculopathy=1	0.0 (0.0-0.9)
**Advanced Diabetic Eye Disease**	2	2 (100.0%)	59.0±5.7	0.30±0.14	None=0<br>Yes=2 (100.0%)<br>Operated=0<br>Not assess=0	None=0<br>Mild=0<br>Mod=0<br>Prepr=0<br>PDR=0<br>Lasered=1 (50.0%)<br>Advanced=1 (50.0%)	69.0±18.4	None=0<br>Yes=1 (50.0%)<br>Operated=1 (50.0%)<br>Not assess=0	Advanced DR=2	0.0

Supplemental Table S12. Baseline age and diabetes duration in patients who completed ≥10 years of follow-up (C) compared with those who did not (NC). Abbreviations: MAD: Mean Absolute Deviation; IQR: Interquartile Range.

Variable	C Number	C Median	C MAD	C IQR	NC Number	NC Median	NC MAD	NC IQR	Mann-Whitney p	Significance ($p<0.05$)
**Age at first screening (years)**	3622	55	9	42.0–62.8	9798	64	8	54.0–71.0	0	YES
**Diabetes duration at first screening (years)**	3622	6	5	2.0–13.0	9798	6	5	1.0–15.0	0.1728	NO

Supplemental Figure S1. Visual acuity (logMAR) at first screening by grading of DR and presence/absence of cataract. Abbreviations: Prol: Proliferative, Preprol: Preproliferative, DR: Diabetic Retinopathy.

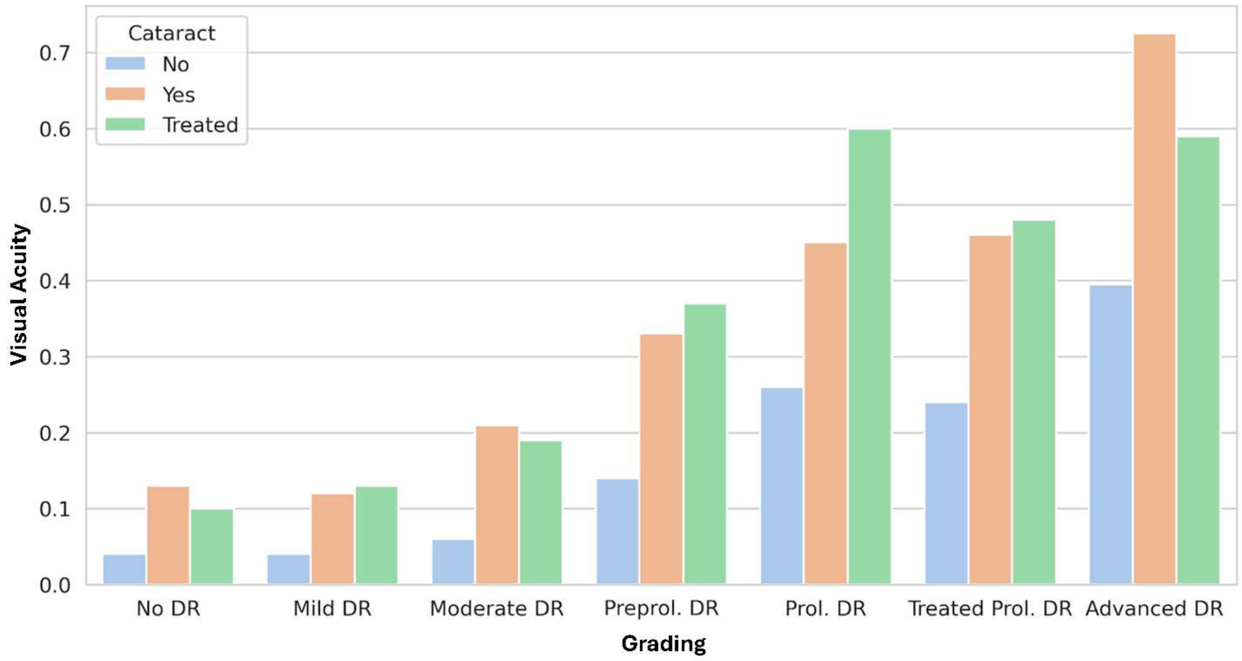

Supplemental Figure S2. Boxplot of visual acuity before and 1,874±1,252 days after photocoagulation for preproliferative or proliferative DR detected at screening.

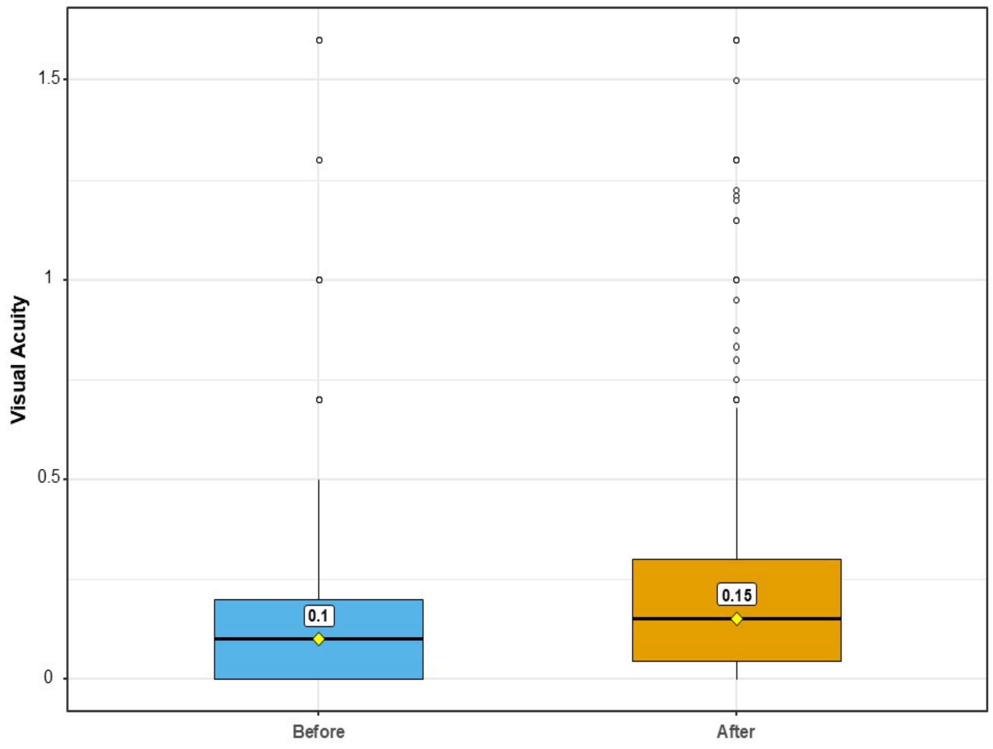

Supplemental Figure S3. Boxplot of visual acuity before and 1,998±1,288 days after focal/grid photocoagulation for diabetic macular edema detected at screening.

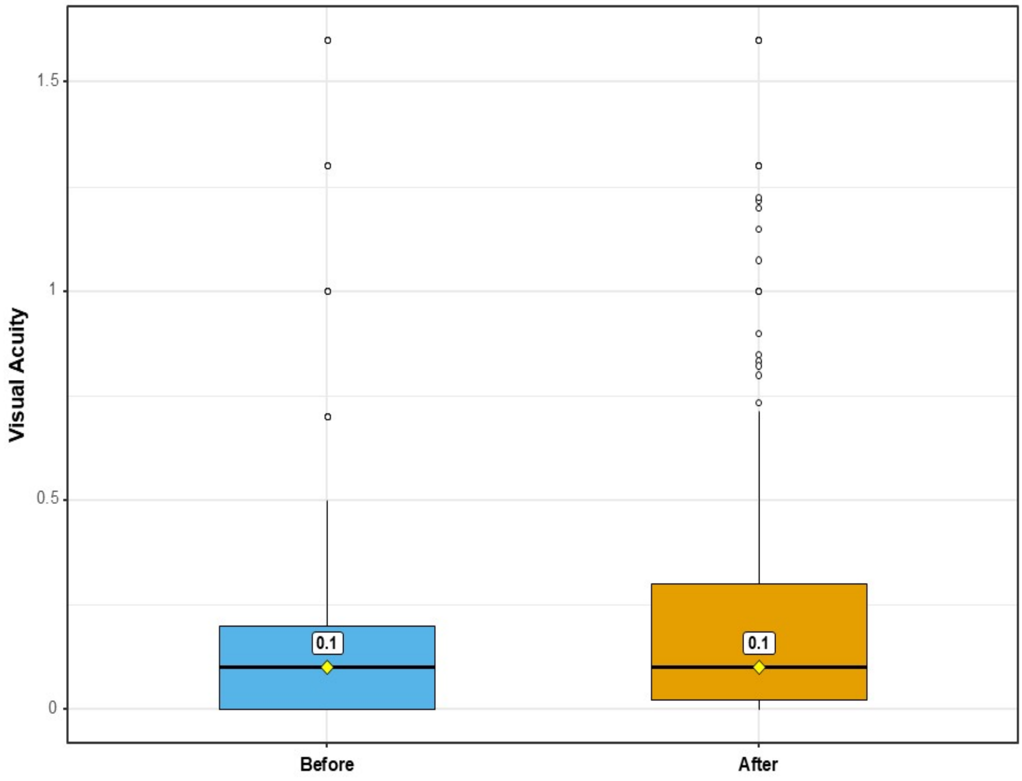

Supplement: Supplementary file 1 [file medsci-14-00355-s001.zip › medsci-4231613-Supplementary.pdf]
